# Supplementary figures and images for: Proteonematalycus wagneri Kethley reveals where the opisthosoma begins in acariform mites
Source: PLoS One. 2022 Feb 25;17(2):e0264358. doi: 10.1371/journal.pone.0264358 (PMC8880937; doi:10.1371/journal.pone.0264358)

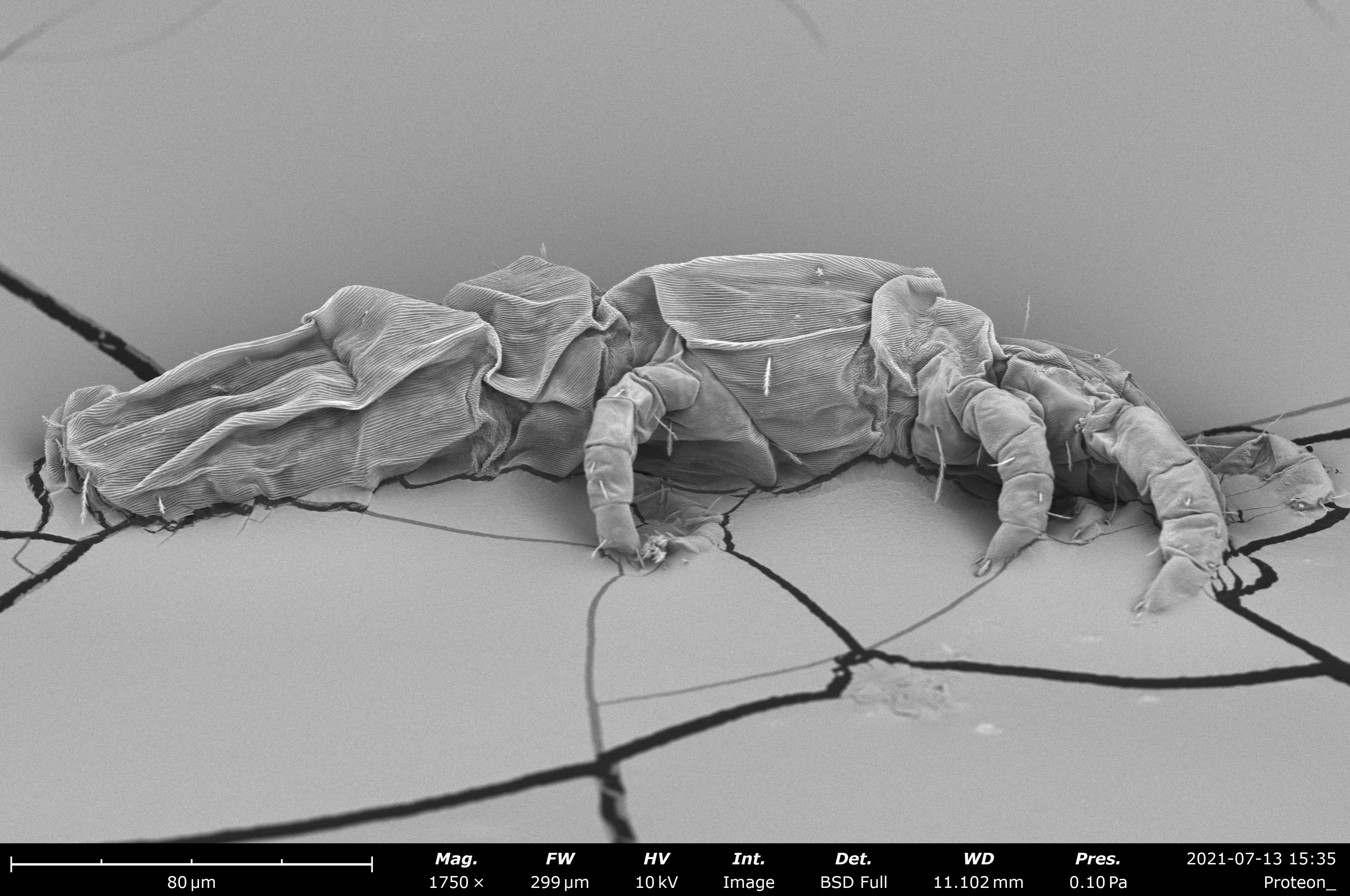

Supplement: S1 Fig — (TIF) [file pone.0264358.s001.tif]

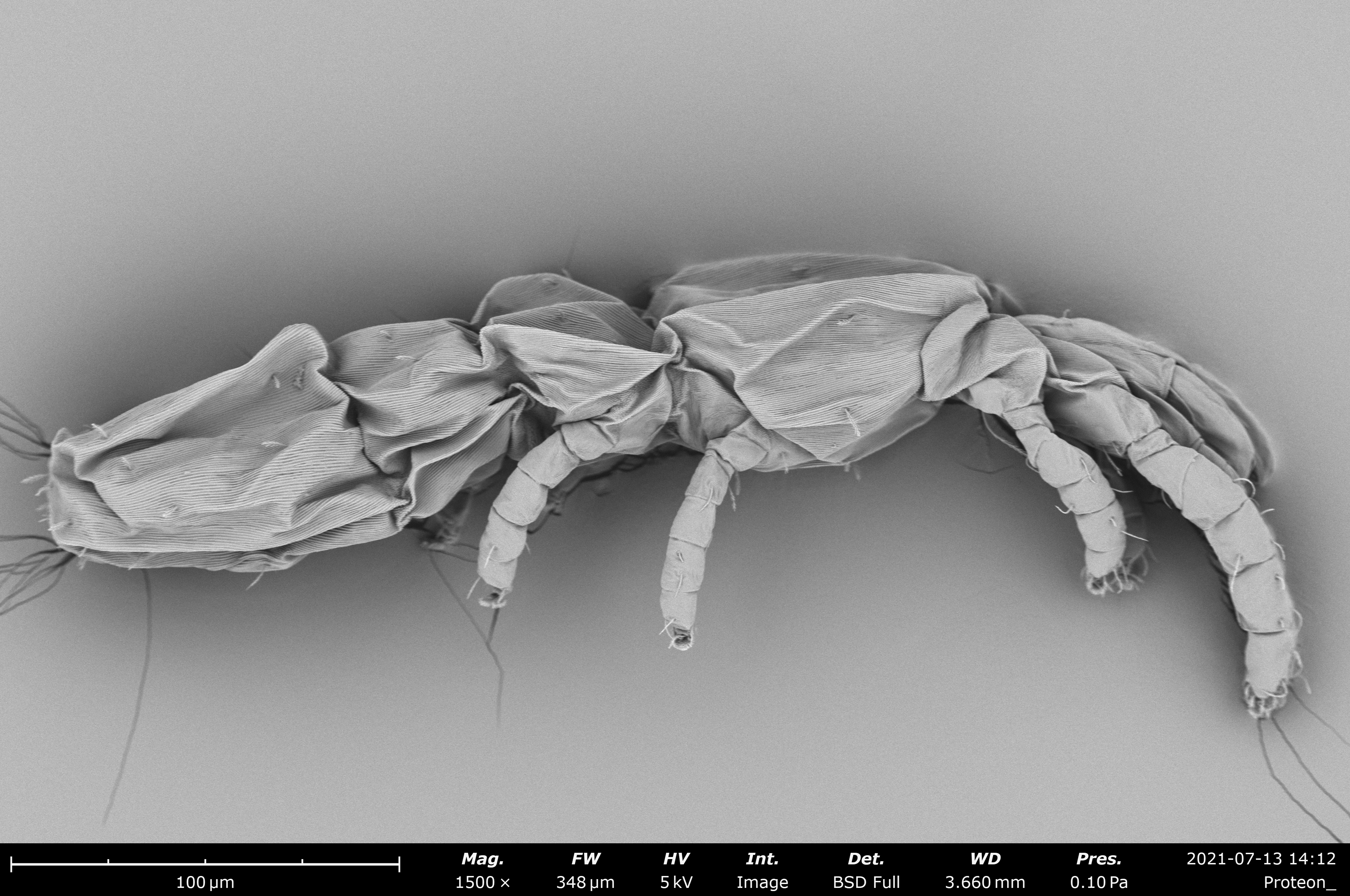

Supplement: S2 Fig — (TIF) [file pone.0264358.s002.tif]

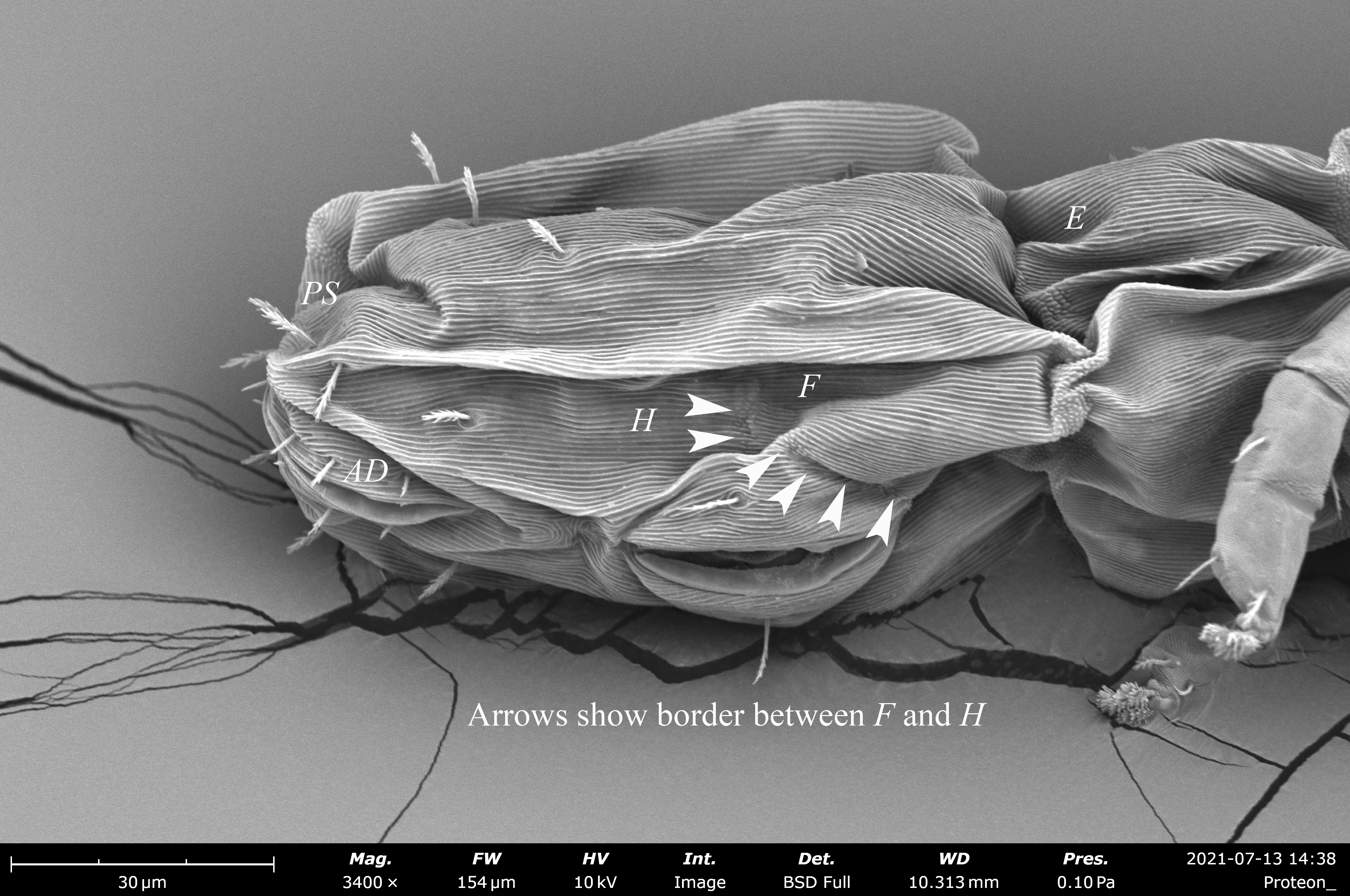

Supplement: S3 Fig — (TIF) [file pone.0264358.s003.tif]

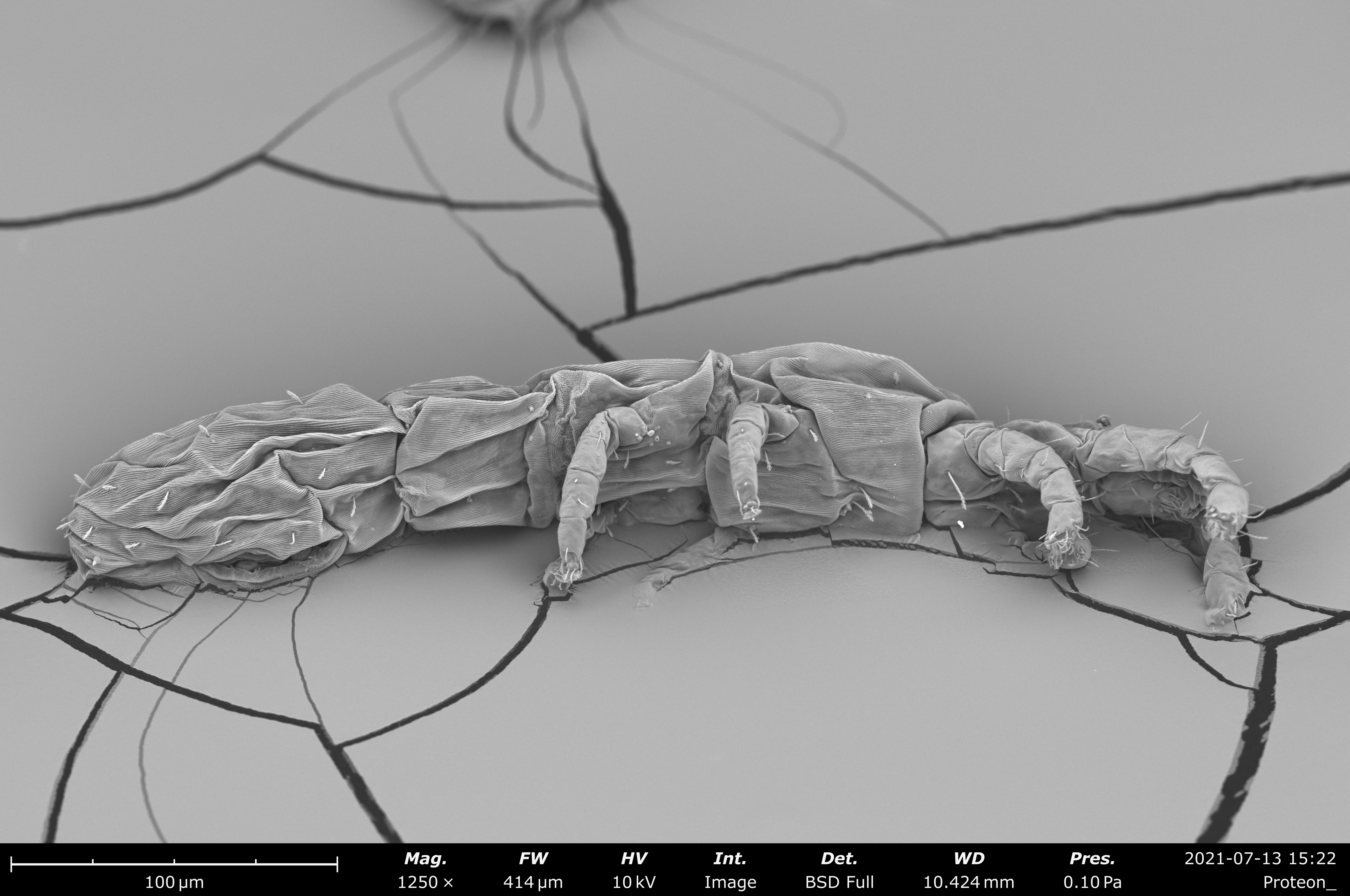

Supplement: S4 Fig — (TIF) [file pone.0264358.s004.tif]

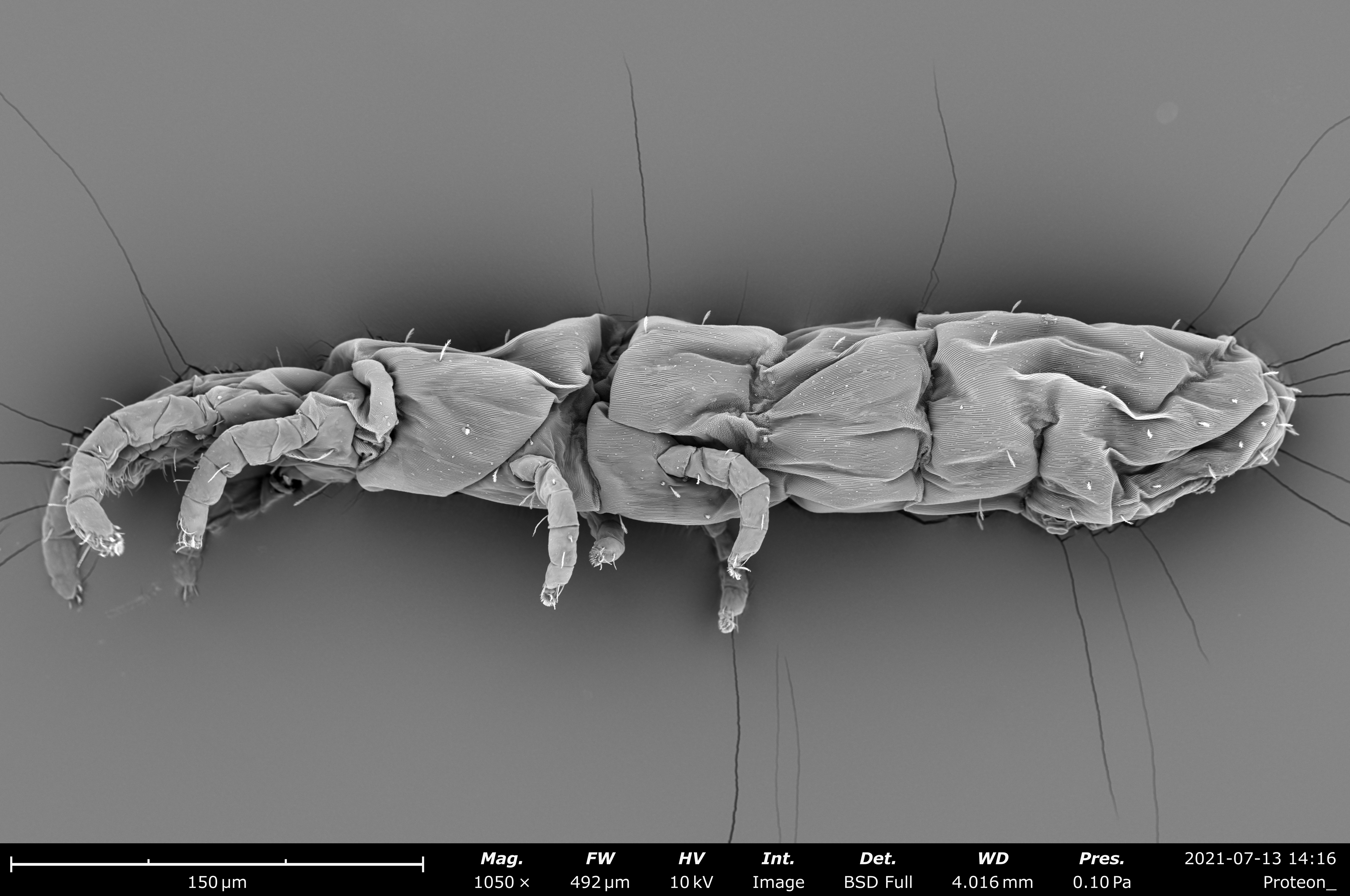

Supplement: S5 Fig — (TIF) [file pone.0264358.s005.tif]

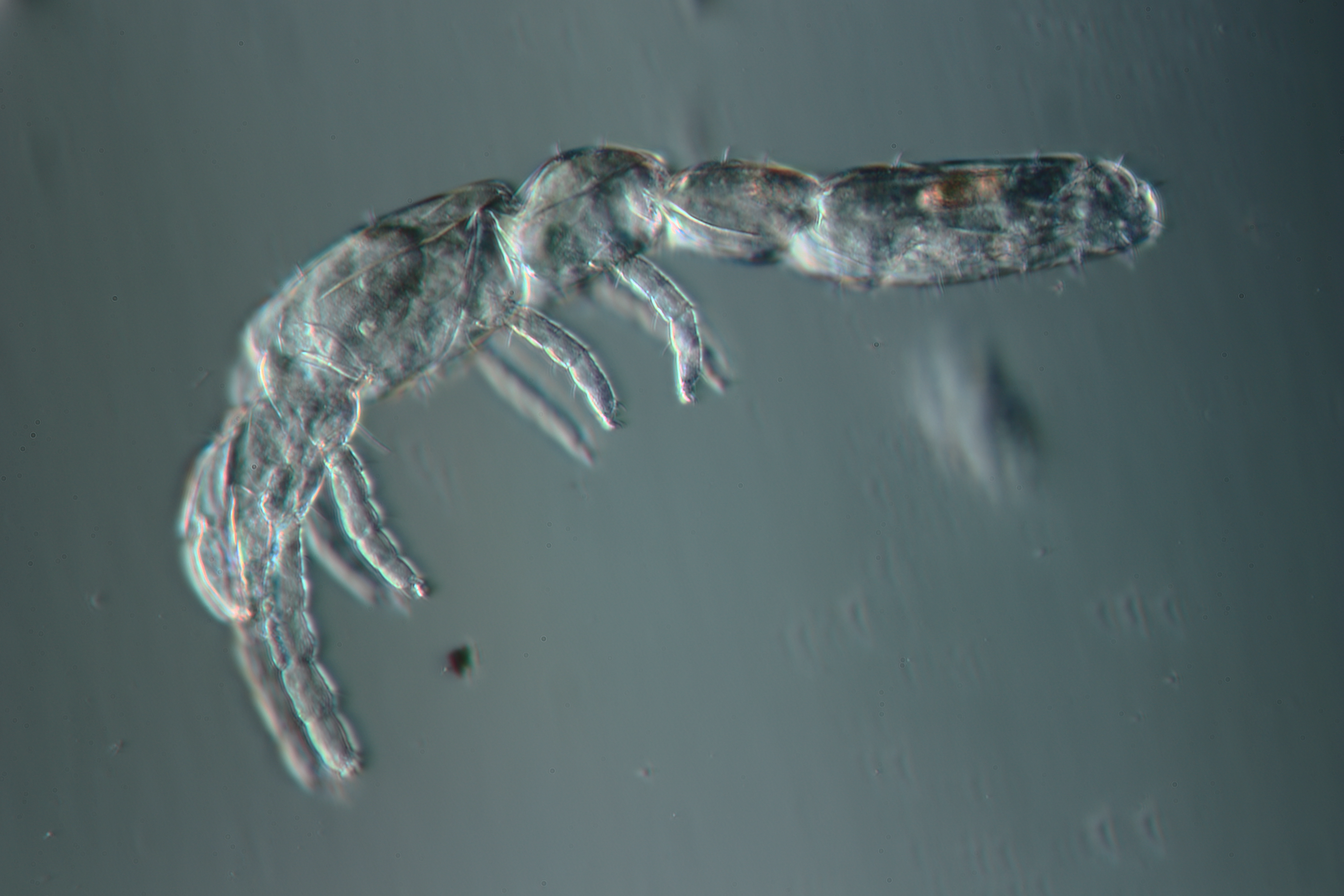

Supplement: S7 Fig — (TIF) [file pone.0264358.s007.tif]
