# Supplementary material for: Proteonematalycus wagneri Kethley reveals where the opisthosoma begins in acariform mites
Source: PLoS One. 2022 Feb 25;17(2):e0264358. doi: 10.1371/journal.pone.0264358 (PMC8880937; doi:10.1371/journal.pone.0264358)
Supplement: Attachment — Submitted filename: PONE-D-21-31633_MS_reviewer comments.pdf [file pone.0264358.s008.pdf]

PLOS ONE

Proteonematalycus wagneri Kethley reveals where the opisthosoma begins in acariform mites

--Manuscript Draft--

Manuscript Number:	PONE-D-21-31633
Article Type:	Research Article
Full Title:	Proteonematalycus wagneri Kethley reveals where the opisthosoma begins in acariform mites
Short Title:	Proteonematalycus wagneri Kethley reveals where the opisthosoma begins in acariform mites
Corresponding Author:	Samuel J Bolton, PhD<br>Florida Department of Agriculture and Consumer Services<br>Gainesville, Florida UNITED STATES
Keywords:	Acariformes; Segmentation; Somite; Opisthosoma; Disjugal Suture; Metapodosoma; Prosoma; Arachnida
Abstract:	It is generally thought that the anterior border of the opisthosoma of acariform mites is delimited by the disjugal suture, but the intersegmental furrows of Proteonematalycus wagneri Kethley instead indicate that this suture intersects the metapodosoma. Accordingly, the anterior border of the opisthosoma is between segments D and E. Therefore, segments C and D are metapodosomal and not opisthosomal. The disjugal suture is probably an apomorphic border that is only present in oribatids. Homology between the disjugal suture and the anterior border of the opisthosoma has never been supported with evidence other than the superficial appearance of tagmosis. The alternative homology can be accommodated by a model in which the proterosoma warps. The metapodosoma can readily undergo elongation because it is not constrained by the warp. This would explain why there is a large interval between legs II and III in most mites with elongate bodies.
Order of Authors:	Samuel J Bolton, PhD
Additional Information:	
Question	Response
**Financial Disclosure**<br><br>Enter a financial disclosure statement that describes the sources of funding for the work included in this submission. Review the submission guidelines for detailed requirements. View published research articles from *PLOS ONE* for specific examples.<br><br>This statement is required for submission and **will appear in the published article** if the submission is accepted. Please make sure it is accurate.	The author received no specific funding for this work.

Powered by Editorial Manager® and ProduXion Manager® from Aries Systems Corporation

**Unfunded studies**<br>Enter: *The author(s) received no specific funding for this work.*<br><br>**Funded studies**<br>Enter a statement with the following details:<br>• Initials of the authors who received each award<br>• Grant numbers awarded to each author<br>• The full name of each funder<br>• URL of each funder website<br>• Did the sponsors or funders play any role in the study design, data collection and analysis, decision to publish, or preparation of the manuscript?<br>• **NO** - Include this sentence at the end of your statement: *The funders had no role in study design, data collection and analysis, decision to publish, or preparation of the manuscript.*<br>• **YES** - Specify the role(s) played.<br><br>* typeset	
**Competing Interests**<br><br>Use the instructions below to enter a competing interest statement for this submission. On behalf of all authors, disclose any competing interests that could be perceived to bias this work—acknowledging all financial support and any other relevant financial or non-financial competing interests.<br><br>This statement is required for submission and **will appear in the published article** if the submission is accepted. Please make sure it is accurate and that any funding sources listed in your Funding Information later in the submission form are also declared in your Financial Disclosure statement.<br><br>View published research articles from *PLOS ONE* for specific examples.	The authors have declared that no competing interests exist.

**NO authors have competing interests**<br><br>Enter: *The authors have declared that no competing interests exist.*<br><br>**Authors with competing interests**<br><br>Enter competing interest details beginning with this statement:<br><br>*I have read the journal's policy and the authors of this manuscript have the following competing interests: [insert competing interests here]*<br><br>* typeset	
**Ethics Statement**<br><br>Enter an ethics statement for this submission. This statement is required if the study involved:<br><br>• Human participants<br>• Human specimens or tissue<br>• Vertebrate animals or cephalopods<br>• Vertebrate embryos or tissues<br>• Field research<br><br>Write "N/A" if the submission does not require an ethics statement.<br><br>General guidance is provided below. Consult the submission guidelines for detailed instructions. **Make sure that all information entered here is included in the Methods section of the manuscript.**	INDU-2013-SCI-004<br>Study no INDU-00358<br>The US National Park Service

Powered by Editorial Manager® and ProduXion Manager® from Aries Systems Corporation

**Format for specific study types**<br><br>**Human Subject Research (involving human participants and/or tissue)**<br>• Give the name of the institutional review board or ethics committee that approved the study<br>• Include the approval number and/or a statement indicating approval of this research<br>• Indicate the form of consent obtained (written/oral) or the reason that consent was not obtained (e.g. the data were analyzed anonymously)<br><br>**Animal Research (involving vertebrate animals, embryos or tissues)**<br>• Provide the name of the Institutional Animal Care and Use Committee (IACUC) or other relevant ethics board that reviewed the study protocol, and indicate whether they approved this research or granted a formal waiver of ethical approval<br>• Include an approval number if one was obtained<br>• If the study involved *non-human primates*, add *additional details* about animal welfare and steps taken to ameliorate suffering<br>• If anesthesia, euthanasia, or any kind of animal sacrifice is part of the study, include briefly which substances and/or methods were applied<br><br>**Field Research**<br><br>Include the following details if this study involves the collection of plant, animal, or other materials from a natural setting:<br>• Field permit number<br>• Name of the institution or relevant body that granted permission	
**Data Availability**<br><br>Authors are required to make all data underlying the findings described fully available, without restriction, and from the time of publication. PLOS allows rare exceptions to address legal and ethical concerns. See the PLOS Data Policy and FAQ for detailed information.	Yes - all data are fully available without restriction

Powered by Editorial Manager® and ProduXion Manager® from Aries Systems Corporation

A Data Availability Statement describing where the data can be found is required at submission. Your answers to this question constitute the Data Availability Statement and **will be published in the article**, if accepted.<br><br>**Important:** Stating 'data available on request from the author' is not sufficient. If your data are only available upon request, select 'No' for the first question and explain your exceptional situation in the text box.<br><br>Do the authors confirm that all data underlying the findings described in their manuscript are fully available without restriction?	
**Describe where the data may be found in full sentences. If you are copying our sample text, replace any instances of XXX with the appropriate details.**<br><br>• If the data are **held or will be held in a public repository**, include URLs, accession numbers or DOIs. If this information will only be available after acceptance, indicate this by ticking the box below. For example: *All XXX files are available from the XXX database (accession number(s) XXX, XXX.).*<br>• If the data are all contained **within the manuscript and/or Supporting Information files**, enter the following: *All relevant data are within the manuscript and its Supporting Information files.*<br>• If neither of these applies but you are able to provide **details of access elsewhere**, with or without limitations, please do so. For example:<br><br>*Data cannot be shared publicly because of [XXX]. Data are available from the XXX Institutional Data Access / Ethics Committee (contact via XXX) for researchers who meet the criteria for access to confidential data.*<br><br>*The data underlying the results presented in the study are available from (include the name of the third party*	All relevant data are within the manuscript and its Supporting Information files.

and contact information or URL).<br>• This text is appropriate if the data are owned by a third party and authors do not have permission to share the data.<br><br>* typeset	
Additional data availability information:	

Powered by Editorial Manager® and ProduXion Manager® from Aries Systems Corporation

Manuscript Click here to access/download;Manuscript;Main_Manuscript.docx

RESEARCH ARTICLE

Proteonematalycus wagneri Kethley reveals where the opisthosoma begins in acariform mites

S. J. Bolton[1]*

1 Florida State Collection of Arthropods, Division of Plant Industry, Florida Department of Agriculture and Consumer Services, Gainesville, Florida, USA

*samuel.bolton77@gmail.com

Abstract

It is generally thought that the anterior border of the opisthosoma of acariform mites is delimited by the disjugal suture, but the intersegmental furrows of *Proteonematalycus wagneri* Kethley instead indicate that this suture intersects the metapodosoma. Accordingly, the anterior border of the opisthosoma is between segments *D* and *E*. Therefore, segments *C* and *D* are metapodosomal and not opisthosomal. The disjugal suture is probably an apomorphic border that is only present in oribatids. Homology between the disjugal suture and the anterior border of the opisthosoma has never been supported with evidence other than the superficial appearance of tagmosis. The alternative homology can be accommodated by a model in which the proterosoma warps. The metapodosoma can readily undergo elongation because it is not constrained by the warp. This would explain why there is a large interval between legs II and III in most mites with elongate bodies.

Introduction

Mites comprise two superorders: Parasitiformes and Acariformes. Almost all mites have lost the furrows that delineate their body segments. However, the dorsal setae of acariform mites are typically arranged in transverse rows, which correspond with the underlying body segments [1]. There are conflicting interpretations concerning the relationship of two of these

segments, *C* and *D* (bearing setae *c* and *d*), to the metapodosoma, which bears legs III and IV (Fig. 1A). Reuter [2] homologized segments *C* and *D* with the metapodosoma. More recently, the same interpretation was put forward by Weigmann [3] (Fig. 1B). Van der Hammen instead homologized segments *C* and *D* with the first two segments of the opisthosoma [4] (Fig. 1C, D), which was based on his hypothesis that the anterior border of the opisthosoma is represented by the disjugal suture (Fig. 1E). Many if not most acarologists accord with van der Hammen's hypothesis by referring to the structures associated with segments *C* and *D* (principally setae and plates) as 'opisthosomal'. The alternative term 'hysterosomal' (the region of the body that is behind the sejugal furrow), is neutral with respect to either hypothesis.

Figure 1. Terminology and models. A. Simplified representation of segmentation in mites, showing the segments in order but without any of the hypothesized modifications in shape. B. Interpretation of Weigmann [3]. C. Interpretation of Grandjean [5]. E. Interpretation of Klompen *et al.* [6] E. The three main body furrows/sutures of acariform mites. Blue = proterosoma; yellow = metapodosoma; brown = opisthosoma; grey = neutral (no homologue applied); Pc = precheliceral region (acron); Ch = chelicera; Pa = palp; LI–LIV = legs I–IV; *C* = segment *C*; *D* = segment *D*; white arrowhead = abjugal; black arrowhead = disjugal; grey arrowhead = sejugal.

Grandjean based a widely cited model on van der Hammen's hypothesis to explain the body segmentation of acariform mites [5]. According to this model, the dorsal region of the podosoma is dramatically reduced, causing the body regions on either side to be pulled into areas that were occupied by the podosoma (Fig. 1C). Klompen *et al.* also adhered to van der Hammen's hypothesis when they suggested an amendment to Grandjean's model [6]. They infer that the whole of the prosoma has warped somewhat evenly (Fig. 1D), which explains how the dorsal surface of the podosoma can be completely diminished. These interpretations are illustrated with an animation, which is accessible via the following link: https://zenodo.org/record/5512807#.YUOPiflKhaQ.

Proteonematalycus wagneri Kethley, a rare species of mite that is only known from sandy habitats, is exceptionally useful for investigating the arrangement of body segments in Acariformes. Unlike other basal acariform mites, in which the furrows that delimit the segments are restricted to the dorsum, some of the furrows of *P. wagneri* completely encircle the hysterosoma, clearly revealing which segments are associated with the metapodosoma.

This mite was examined with a scanning electron microscope (SEM) and a light microscope in order to confirm that these furrows are intersegmental.

Results

The integument of *P. wagneri* is extremely soft and fragile, causing it to readily distort when removed from alcohol. Alcohol stored specimens of this species completely shriveled up when viewed with cryo-SEM, which has been used with a high degree of success on other soft-bodied mites [7]. By comparison, mites that were desiccated using ethanol–hexamethyldisilazane (HMDS) provided relatively good images using conventional SEM, although there was still some shriveling (Fig. 2A, B). SEM reveals that each hysterosomal segment is clearly delimited by intersegmental furrows, which fall on either side of a transverse row of setae. A closeup of the integument shows that the striae break up into very fine protuberances along the base of each furrow (Fig. 2B). By uncovering furrows that correspond with the borders of all the hysterosomal segments, SEM removes any remaining ambiguity concerning the homology of the furrows with segmental borders; the drawings in the original species description do not include the furrow between segments *F* and *H* [8].

Figure 2. *Proteonematalycus wagneri* Kethley. A. Lateral view of female (SEM; FSCA 00030222). B. Lateral view of segments *F–AN* (SEM; same female as above). C. Lateral view of female (light microscopy; FSCA 00030224), approximately 2 minutes after immersion in PVA. White dotted lines delimit segments *PS–AN*. Small white arrowheads delimit the dorsolateral borders of coxae III and IV (note that there is slight sagging of the integument over the border of coxa III).

Despite the relatively low image resolution, light microscopy demonstrates how *P. wagneri* appears when it has not undergone any shriveling. This species reveals very distinct intersegmental furrows (Fig. 2C). This is an important distinction from mites that exhibit a disjugal suture [9], which can be explained as a non-segmental feature because it bears no real resemblance to an intersegmental furrow. A suture intersects a plane whereas an intersegmental furrow is a slight constriction between two segmental humps (Fig. 3A). Intersegmental furrows are clearly exhibited in other basal groups of mites that are known to exhibit body segments [6,10,11].

Figure 3. The metapodosomal-opisthosomal boundary. A. Illustration of the difference between a furrow and a suture. B. Same model as Fig. 1E, but color coded to show that the disjugal suture intersects the metapodosoma

(yellow). C. A new model for explaining elongate bodied acariform mites. Blue = proterosoma; yellow = metapodosoma; brown = opisthosoma; Fu = furrow; Su = suture; black arrowhead = disjugal suture.

With respect to segmental homology, *P. wagneri* shows that segments *C* and *D* bear legs III and IV, respectively, and so these segments represent the metapodosoma, whereas segment *E* represents the first segment of the opisthosoma. There is no trace of a disjugal suture or furrow.

Discussion

The disjugal suture

Other than the superficial appearance of tagmosis in some oribatids [4,5], there is no evidence that the disjugal suture forms a boundary between the metapodosoma and opisthosoma. This suture must instead intersect the metapodosoma (Fig.3B). Perhaps the main reason that van der Hammen's hypothesis has been so widely adopted is that it adds two additional segments to the acariform body (segments *C* and *D* are treated as separate from the metapodosoma), which makes the body segment count of this lineage closer to that of other arachnids [4]. However, this is not a strong argument because there is noticeable variation in the number of body segments among other arachnids; Scorpiones have nineteen body segments, whereas Opiliones have only fifteen [1].

Based on van der Hammen's hypothesis, the term 'disjugal' is often applied to the dorsal part of the sejugal furrow of non-oribatid taxa [12–15]. And in *Neognathus*, longitudinal ridges of tubercles have been mislabeled as disjugal (each ridge cuts between setae *c1* and *c2* and so cannot delineate the opisthosoma) [16]. But a true disjugal suture is probably only present in Oribatida. This lineage has evolved a suite of defensive modifications that are found in association with the disjugal suture [17,18]. It is relatively common for secondarily derived borders to form in association with sclerotization. For example, labidostomatids have a distinctive groove that divides the body into a dorsal and ventral shield [19]. Therefore, it seems likely that the disjugal suture originated as an apomorphic border as a result of sclerotization in oribatids.

The presence of visible body segments, involving intersegmental furrows, is very obvious in some soft-bodied basal acariform taxa [10,11]. This is also the case in other basal arachnid lineages, for example, Mesothelae within Araneae [20,21], and Opilioacarida within Parasitiformes [6,22]. It is therefore appropriate to base the interpretation of acariform body segmentation on *P. wagneri*, which is a basal acariform mite with a complete set of intersegmental furrows along the hysterosoma.

A disjugal furrow is clearly absent from *P. wagneri*. There appears to be no example of a true disjugal furrow, as opposed to a disjugal suture, in any mite (Fig. 3A). Instead, the intersegmental furrows of all mites that exhibit them are concordant with Reuter [2] and Weigmann's [3] interpretation of where the opisthosoma begins. Accordingly, the border between the metapodosoma and the opisthosoma is formed by a transverse furrow between segments *D* and *E* (Fig. 2A). This is shown by *P. wagneri*. Mites in the family Tarsocheylidae also show this, although they have an incomplete set of intersegmental furrows along the hysterosoma [23]. And these taxa affirm what has already been inferred from other taxonomic groups, namely Siteroptidae [2] and Oribatida [3].

Model

The relatively posterior position of the paired eyes of mites has been explained by the warping of the entire prosoma [6] (Fig. 1D). However, *P. wagneri* indicates that the metapodosoma cannot be part of the warp. B[illegible]arping of the proterosoma still seems likely based on the position of the paired eyes. This would also explain the uneven distribution of legs along the bodies of mites in which segment *C* has elongated (in most elongate bodied taxa this segment elongates more than any other). Legs I and II are alw[illegible] tightly packed with the palps and chelicerae [23–25] (Fig. 2), which is probably because of a constraint attributable to a proterosomal warp (Fig 3C). But legs III and IV are much more posterior because they belong on segments *C* and *D*. These segments can readily elongate because they are not constrained by that warp. It is noteworthy that, primitively, the euarthropod body underwent tagmosis into a head (proterosoma) and trunk (hysterosoma) [26,27]. Warping of the proterosoma may in some way be linked to this tagmosis (Fig. 3C).

Materials and methods

Collection

Proteonematalycus wagneri was collected from foredune sand using heptane flotation [28]. Collection event: U.S.A., Indiana, Porter Co., Indiana Dunes State Park, 41.6780 N 87.0081 W, sand dune (10 cm deep); collector: Samuel Bolton, 16 May, 2013 (FSCA 00030222: Female x1, Deutonymph x1, Protonymph x1, larva x1) (FSCA 00030224: Female x1; FSCA 00030225: Deutonymph x1).

SEM

Specimens of *P. wagneri* were transferred from a storage medium of 95% ethanol into the following series of solvents: 1) absolute ethanol; 2) 50:50 volume HMDS; 3) 100% HMDS. Immersion in each fluid medium lasted approximately five minutes. A minuten pin, which had been glued onto a FisherbrandTM plain wooden applicator, was used to maneuver the specimens between solutions. For the final step, the HMDS was left to evaporate. The specimens were then mounted on SEM stubs and sputter coated with approximately 70 nm of gold/palladium using a Denton IV sputtercoater. Micrographs were captured with a Phenom XL G2 Desktop SEM.

Light microscopy

Examination was with a compound microscope (Leica DM2500) equipped with differential interference contrast (DIC) and a digital SLR (Canon EOS 80D). Imaging was with a dry, 10x objective (brightfield). Polyvinyl alcohol (PVA) mounting medium was used to hold the specimens in a relatively still position. Specimens were imaged without a coverslip within minutes of being placed into the PVA. Contrast was heightened by setting the turret of the DIC between the 10 and 40 intervals.

Acknowledgements

Jonathan Bremer, at the Florida State Collection of Arthropods, undertook the SEM imaging of *P. wagneri* (Fig. 2A, B). The U.S. National Park Service granted the author permission to collect from the Indiana Dunes National Lakeshore. The following individuals are thanked for their contribution through internal review and/or useful discussion: Erin Powell, Elijah Talamas, Paul Skelley (Florida Department of Agriculture and Consumer Services, Gainesville, Florida, USA) and Hans Klompen (Ohio State University, Columbus, Ohio, USA). The Florida Department of Agriculture and Consumer Services – Division of Plant Industry are thanked for their support on this contribution.

References

1. Dunlop JA, Lamsdell JC. Segmentation and tagmosis in Chelicerata. Arthropod Struct Dev. 2017;46: 395–418. doi:10.1016/j.asd.2016.05.002
2. Reuter E. Zur Morphologie und Ontogenie der Acariden mit besonderer Berücksichtigung von Pediculopis graminum. Acta Soc Sci Fenn. 1909;36: 1–288.
3. Weigmann G. The body segmentation of oribatid mites from a phylogenetic perspective. In: Halliday RB, Walter DE, Proctor HC, Norton RA, Colloff MJ, editors. Acarology: Proceedings of the 10th International Congress. Melbourne: CSIRO; 2001. pp. 43–49.
4. Van der Hammen L. The addition of segments during the postembryonic ontogenesis of the Actinotrichida (Acarida) and its importance for the recognition of the primary subdivision of the body and the original segmentation. Acarologia. 1963;5: 443–454.
5. Grandjean F. Stases. Actinopiline. Rappel de ma classification des acariens en 3 groupes majeurs. Terminologie en soma. Acarologia. 1969;11: 796–827.
6. Klompen H, Vázquez MM, Bernardi LF de O. Post-embryonic development in the mite suborder Opilioacarida, with notes on segmental homology in Parasitiformes (Arachnida). Exp Appl Acarol. 2015;67: 183–207. doi:10.1007/s10493-015-9939-7
7. Bolton SJ, Klompen H, Bauchan GR, Ochoa R. A new genus and species of Nematalycidae (Acari: Endeostigmata). J Nat Hist. 2014;48: 1359–1373. doi:10.1080/00222933.2013.859318
8. Kethley J. Proteonematalycidae (Acari: Acariformes), a new mite family from fore-dune sand of Lake Michigan. Int J Acarol. 1989;15: 209–217. doi:10.1080/01647958908683851
9. Alberti G, Coons LB. Vol. 8C. Chelicerate Arthropoda. Acari: Ticks. In: Harrison FW, Rainer FF, editors. Microscopic Anatomy of Invertebrates. New York: John Wiley & Sons; 1999. pp. 515–1265.
10. Walter DE. Endemism and cryptogenesis in “segmented” mites: A review of Australian Alicorhagiidae, Terpnacaridae, Oehserchestidae and Grandjeanicidae (Acari: Sarcoptiformes). Aust J Entomol. 2001;40: 207–218. doi:10.1046/j.1440-6055.2001.00226.x
11. Uusitalo M. Revision of the family Alycidae (Acariformes, Acari), with special reference to European species. Department of Biological and Environmental Sciences. University of Helsinki. 2010.
12. Alzuet AB, Delgado AGC. Pachygnathidae, a new family of mites belonging

to argentine fauna. (Prostigmata). Rev la Soc Entomológica Argentina. 1984;43: 85–90.
13. Olivier PAS, Theron PD. A new species of Speleorchestes (Nanorchestidae: Prostigmata) from a savanna biotope in South Africa. South African J Zool. 1989;24: 356–360. doi:10.1080/02541858.1989.11448177
14. Jesionowska K. A morphological study of the genus Penthalodes (Acari, Prostigmata, Eupodoidea, Penthalodidae) with description of a new species. Zootaxa. 2010; 29–49. doi:10.11646/zootaxa.2672.1.2
15. Zacharda M, Isaia M, Piva E. New troglobitic species of the genus Troglocheles (Acari: Prostigmata: Rhagidiidae) from caves in northern Italy and Austria, with a key to adult species of the genus. J Nat Hist. 2011;45: 641–666. doi:10.1080/00222933.2010.535914
16. Doğan S, Doğan S. New record of an unusual caligonellid (Acari: Caligonellidae) species in Turkey: Neognathus eupalopus Meyer Ueckermann, with the description of a new species. Syst Appl Acarol. 2019;24: 1162–1176. doi:10.11158/saa.24.7.5
17. Schmelzle S, Helfen L, Norton RA, Heethoff M. The ptychoid defensive mechanism in Euphthiracaroidea (Acari: Oribatida): A comparison of exoskeletal elements. Soil Org. 2008;80: 233–247.
18. Schmelzle S, Helfen L, Norton RA, Heethoff M. The ptychoid defensive mechanism in Phthiracarus longulus (Acari, Oribatida, Phthiracaroidea): Exoskeletal and muscular elements. Soil Org. 2010;82: 253–273.
19. Crossley DA, Simmons BL. Akrostomma gabriella n. sp. from Southern California, with Comments on the Genus (Acari: Labidostomatidae). Proceedings of the Entomological Society of Washington. 2015. pp. 458–462. doi:10.4289/0013-8797.117.4.458
20. Xin X, Liu F, Chen J, Ono H, Li D, Kuntner M. A genus-level taxonomic review of primitively segmented spiders (Mesothelae, Liphistiidae). Zookeys. 2015;488: 121–151. doi:10.3897/zookeys.488.8726
21. Garrison NL, Rodriguez J, Agnarsson I, Coddington JA, Griswold CE, Hamilton CA, et al. Spider phylogenomics: untangling the Spider Tree of Life. PeerJ. 2016;2016: e1719. doi:10.7717/peerj.1719
22. Klompen H, Lekveishvili M, Black IV WC. Phylogeny of parasitiform mites (Acari) based on rRNA. Mol Phylogenet Evol. 2007;43: 936–951. doi:10.1016/j.ympev.2006.10.024
23. Fuangarworn M, Butcher BA. Two new species of tarsocheylid mites (Acari: Heterostigmata, Tarsocheylidae) from coastal grassland soil in Thailand. Syst Appl Acarol. 2016;21: 255–266. doi:10.11158/saa.21.2.9
24. Bolton SJ, Bauchan GR, Ochoa R, Pooley C, Klompen H. The role of the integument with respect to different modes of locomotion in the Nematalycidae (Endeostigmata). Exp Appl Acarol. 2015;65: 149–161. doi:10.1007/s10493-014-9857-0
25. Khaustov AA. Review of the Paratydeidae (Acari: Prostigmata), with description of three new species. Zootaxa. 2017. pp. 151–212. doi:10.11646/zootaxa.4303.2.1
26. Scholtz G. Cleavage, germ band formation and head segmentation: the ground pattern of the Euarthropoda. In: Fortey RA, Thomas RH, editors. Arthropod Relationships The Systematics Association Special Volume Series. Dordrecht: Springer; 1998. pp. 317–332. doi:10.1007/978-94-011-4904-4_24
27. Waloszek D, Chen J, Maas A, Wang X. Early Cambrian arthropods - New insights into arthropod head and structural evolution. Arthropod Struct Dev. 2005;34: 189–205. doi:10.1016/j.asd.2005.01.005
28. Kethley J. A procedure for extraction of microarthropods from bulk soil samples with emphasis on inactive stages. Agric Ecosyst Environ. 1991;34:

193–200. doi:10.1016/0167-8809(91)90105-7

Figure 1

Click here to access/download;Figure;Fig1.tif

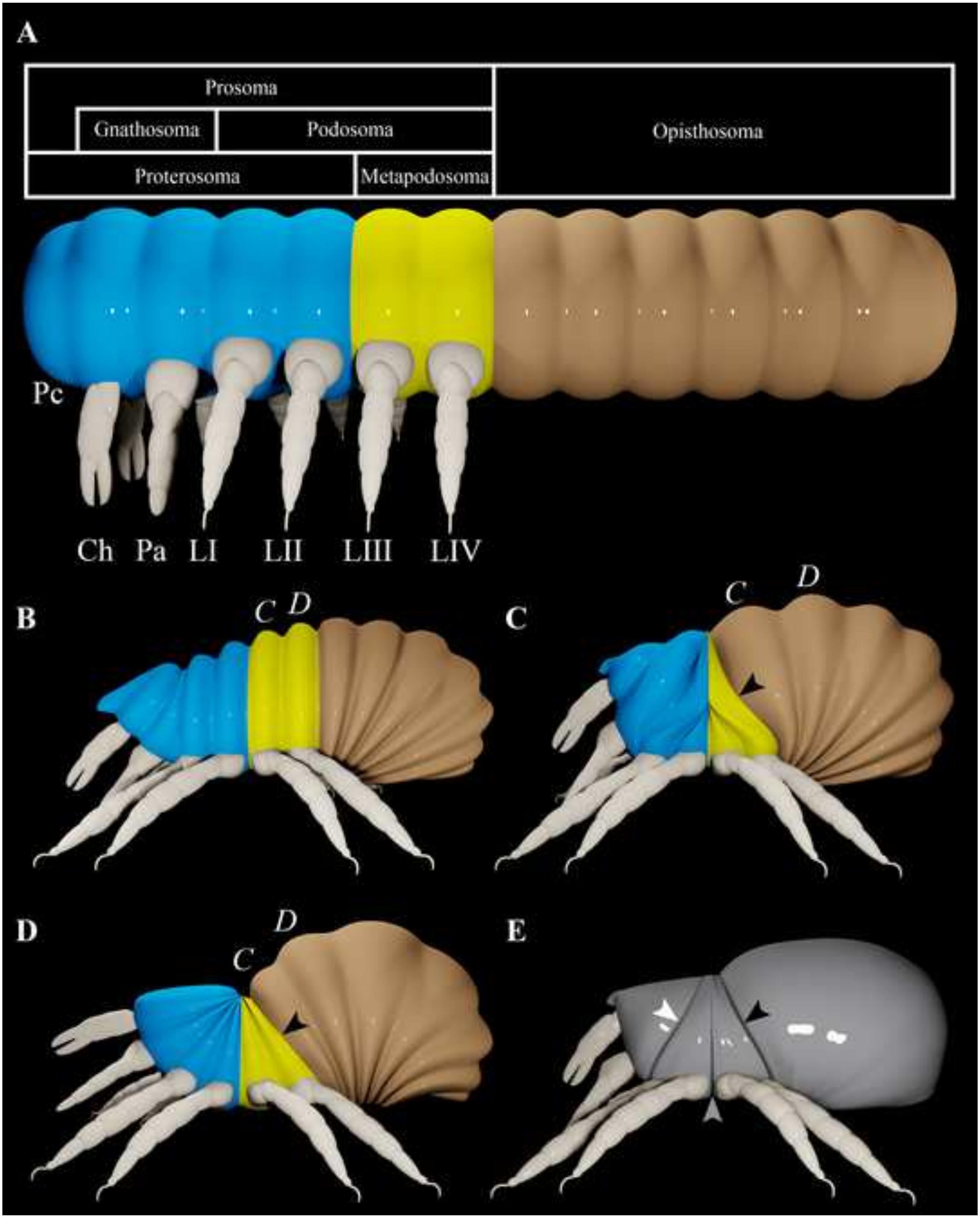

Figure 2

Click here to access/download;Figure;Fig2.tif

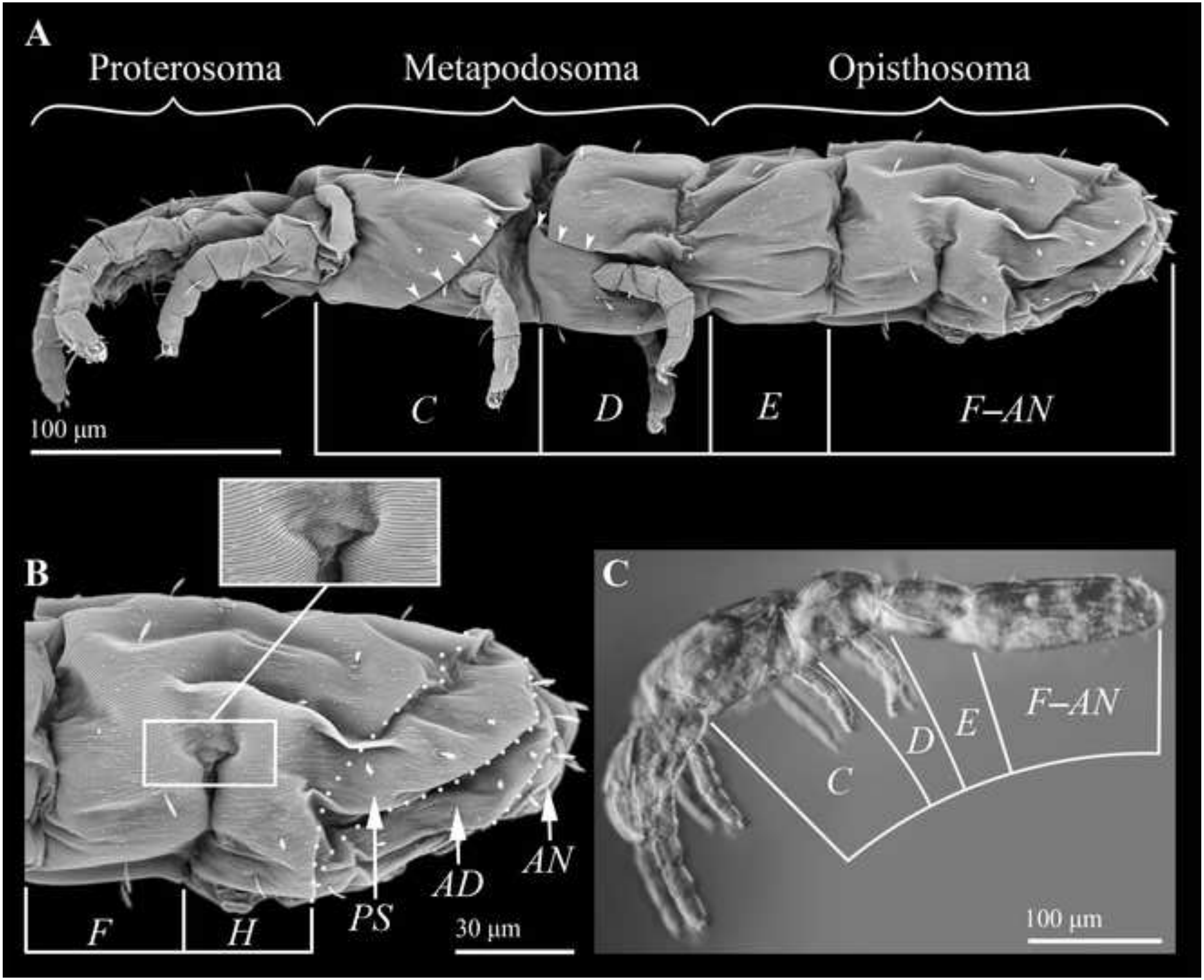

Figure 3 Click here to access/download;Figure;Fig3.tif

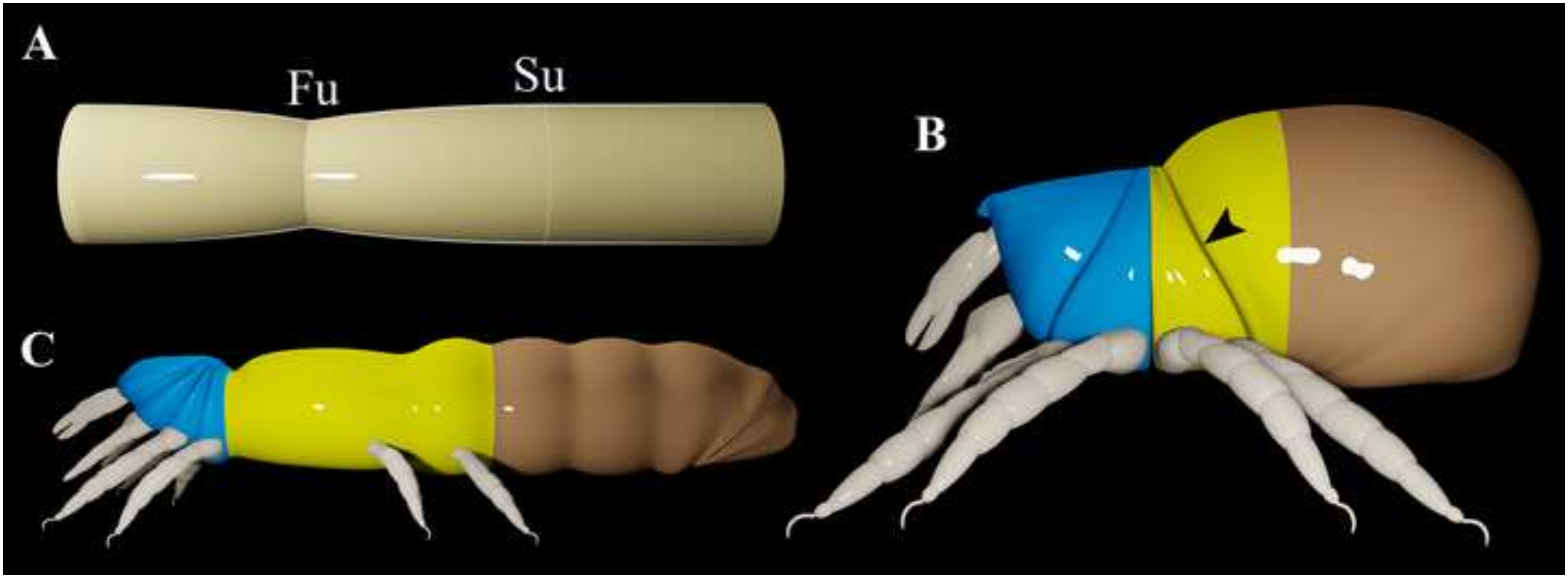

Click here to access/download
Supporting Information
Sup1_FSCA_00030222_LarvaA.jpg

Supplementary

Click here to access/download
Supporting Information
Sup2_FSCA_00030222_LarvaB.jpg

Supplementary

Click here to access/download
Supporting Information
Sup3_FSCA_00030222_Protonymph.jpg

Supplementary

Click here to access/download
Supporting Information
Sup4_FSCA_00030222_Protonymph_E_to_AD.jpg

Supplementary

Click here to access/download
Supporting Information
Sup5_FSCA_00030222_Deutonymph.jpg

Supplementary

Click here to access/download
Supporting Information
Sup6_FSCA_00030225_Deutonymph_8_minutes_after_immersion.jpg
